# Suprasellar Plasmacytoma Leading to the Diagnosis of Multiple Myeloma

**DOI:** 10.7759/cureus.25831

**Published:** 2022-06-10

**Authors:** Joseph T Johnson, Pooja N Bhakta, Ramya D Vinnakota, Bernard Karnath, Maurice Willis

**Affiliations:** 1 Internal Medicine, University of Texas Medical Branch at Galveston, Galveston, USA; 2 Oncology, University of Texas Medical Branch at Galveston, Galveston, USA; 3 Oncology, MD Anderson Cancer Center, Houston, USA

**Keywords:** suprasellar tumor, plasma cell tumor, suprasellar mass, multiple myeloma, intracranial plasmacytoma

## Abstract

Plasmacytomas are a collection of plasma cells that occur as a solitary lesion or in conjunction with multiple myeloma. Intracranial location is uncommon but should be considered as management differs. Plasmacytomas in the suprasellar region are rare but should be considered in the differential diagnosis of suprasellar masses. Clinical presentation and imaging findings have similarities and overlap between pituitary adenomas and plasmacytomas, so the diagnosis depends on biopsy and pathological evaluation. Immunohistological staining is often necessary due to structural similarities to adenomas. Isolated cases may be treated with radiation alone and surgery is reserved for symptoms due to mass effect. Systemic therapy is given if there is evidence of multiple myeloma. In this case report, we present a 52-year-old male who presented with worsening blurry vision associated with headaches and epistaxis of four months duration. CT of the head showed a large mass involving the sella and skull base. Labs showed normal calcium, creatinine, and intact pituitary function. Biopsy of the mass was initially diagnosed as a pituitary adenoma but repeat pathology revealed plasmacytoma. Body imaging revealed diffuse lytic lesions. Bone marrow biopsy and serum electrophoresis were consistent with a diagnosis of multiple myeloma. The patient underwent radiation therapy to the suprasellar mass followed by systemic therapy for multiple myeloma with bortezomib, lenalidomide, and dexamethasone. The patient achieved a very good partial response.

## Introduction

Multiple myeloma and plasmacytomas are plasma cell neoplasms that can occur independently or together. Multiple myeloma makes up 1.8% of all new cases of cancer in the United States. It more commonly affects men and specifically African American men. The median age of diagnosis is 69 years [1]. With the introduction of new therapies, including proteasome inhibitors and autologous stem cell transplants, the five-year relative survival of multiple myeloma has increased dramatically in recent years [2]. Presentation of multiple myeloma can be variable among patients and diagnosis requires careful evaluation of symptoms and laboratory and pathological evaluations.

Plasmacytomas are a collection of malignant plasma cells. If systemic symptoms/spread is present, multiple myeloma is diagnosed. Plasmacytomas are typically further classified into solitary bone plasmacytomas and solitary extramedullary plasmacytomas. Solitary bone plasmacytomas occur in the bone while solitary extramedullary plasmacytomas most often are seen involving the soft tissues of the head, neck, respiratory tract, and gastrointestinal systems without boney involvement and are less common [3].

Patients with plasmacytomas of the sellar region often present with headaches, cranial nerve deficits, visual disturbances, bloody nasal discharge, and eye and craniofacial pain [4]. A biopsy is necessary to establish the diagnosis. Assessment for systemic illness should be pursued upon diagnosis to further guide treatment as 25-45% of patients go on to be diagnosed with multiple myeloma according to one study [5,6].

## Case presentation

A 52-year-old male with a medical history of rheumatoid arthritis presented with a chief complaint of visual changes of four months duration associated with headaches, epistaxis, and fatigue. He was initially seen at another medical facility and imaging of the head revealed a large soft tissue mass. Transsphenoidal subtotal resection of the mass was attempted and halted due to significant blood loss during the surgery. He had improvement in visual symptoms but had persistent epistaxis. The pathology of this initial specimen revealed a diagnosis of pituitary adenoma with negative stains for prolactin, adrenocorticotropic hormone (ACTH), and growth hormone.

**Figure 1:**
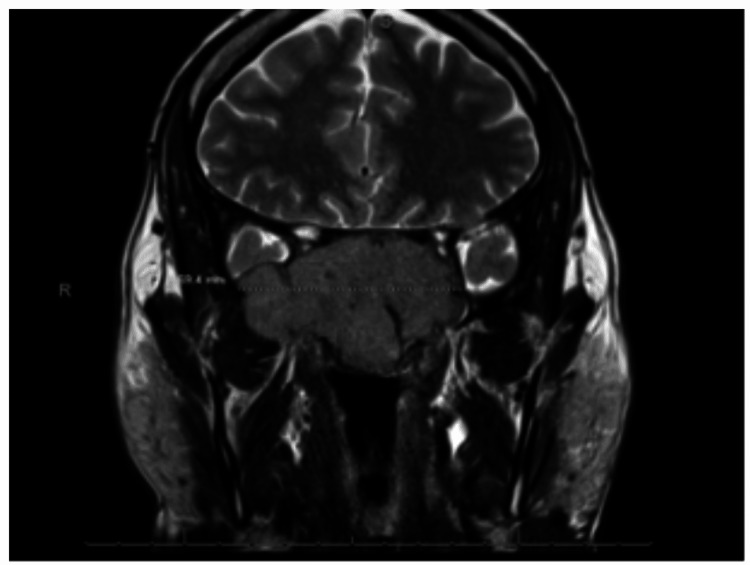
Pituitary magnetic resonance imaging (MRI) MRI depicting a large 7-cm skull base sellar suprasellar mass.

Within weeks from his subtotal resection, visual symptoms recurred with rapid worsening with persistent epistaxis. He was transferred to the current medical facility where a CT of the head showed a 6.4 x 7.1 cm destructive soft tissue mass involving the sella and skull base. MRI of the head showed similar findings as well as invasive features such as mass effect on optic chiasm, invasion into the cavernous sinuses displacement of internal carotid arteries, extension into the right middle cranial fossa, sphenoid sinuses, posterior ethmoid air cells, and clivus, and extension into the prepontine cistern adjacent to the basilar artery (Figure 1). Lab work revealed normal calcium, creatinine, total protein, and serum hormone levels. Prolactin was slightly elevated at 27.4 ng/ml. Due to the invasive and aggressive nature of the mass, the above presentation was thought to be atypical for a pituitary adenoma.

**Figure 2:**
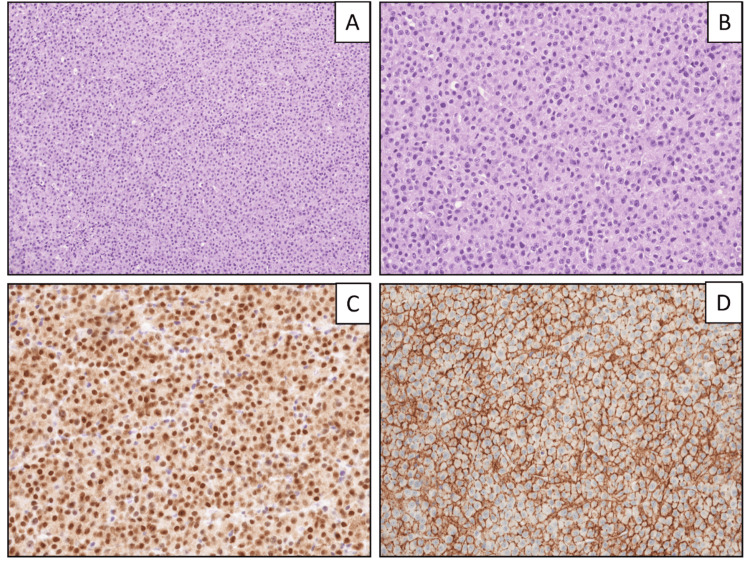
Histology of plasma cells Histological sections show fibroconnective tissue, which is diffusely involved by sheets of plasma cells (A, hematoxylin and eosin (H&E), 100x). Predominant plasma cells are small and mature-appearing but a few large plasma cells have mildly irregular nuclear contours (B, H&E, 200x). Immunohistochemical stains were performed and showed multiple myeloma oncogene-1 (MUM1) (C, 200x) and CD138 (D, 200x) stains, which highlight diffuse sheets of plasma cells.

**Figure 3:**
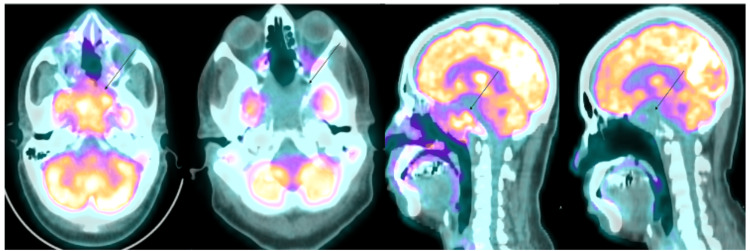
Positron emission tomography (PET) scan Right skull base with standardized uptake value (SUV) of 6.9 before treatment and significantly decreased activity after treatment with radiation and systemic therapy.

Due to persistent symptoms, the patient underwent another resection, which ultimately revealed a diagnosis of plasmacytoma. Due to the plasmacytoid morphological appearance of the cells, additional stains (CD138, lambda light chain, multiple myeloma oncogene-1 (MUM1), CD79a, Ki67) were done, which established the diagnosis of a plasmacytoma (Figure 2). Further workup included systemic staging (Figure 3), which showed multiple lytic lesions and bone marrow involvement (30-40% plasma cells, positive for CD56 and CD117) of plasma cell neoplasm. Myeloma labs showed an M spike of 1.7 g/dl in the gamma region. He had normocytic anemia, which was partially attributed to blood loss from surgeries but there was no hypercalcemia or renal failure. He met the criteria for the diagnosis of multiple myeloma per the International Myeloma Working Group (IMWG). Fluorescence in situ hybridization (FISH) analysis revealed high-risk cytogenetics with the presence of translocation 4:14. The remaining high-risk features were negative including 1q gain, 9p gain, 11q gain, 17p deletion, and t(11;14), t(14;16), or t(14;20) translocations.

The patient then underwent radiation therapy to the base of the skull to target residual mass. He also underwent palliative radiation to target painful areas including his right arm and left hip. He was then started on the standard of care systemic therapy. Due to logistical challenges, he was initially started on CyBorD (cyclophosphamide, bortezomib, and dexamethasone) and later switched to vRD (bortezomib, lenalidomide, and dexamethasone). He tolerated the treatment well and responded well. Per IMWG criteria, he was able to achieve a very good partial response (VGPR) and is currently undergoing evaluation for autologous stem cell transplantation.

## Discussion

The differential for suprasellar masses is broad and includes adenomas (most common), meningiomas, aneurysms, infection, and plasmacytomas among many others. In one study examining the diagnosis of suprasellar masses at an institution over a roughly 11-year period, one out of 2598 masses was diagnosed as a plasmacytoma [7]. Even among patients with multiple myeloma, plasmacytomas are uncommon with a rate of about 3% in one study and 29% in another [5,8]. Although less common, plasmacytoma should be considered in the differential of a suprasellar mass, as it would change treatment. If the mass is found to be a plasmacytoma, further workup should be done as around 25-45% of individuals with a plasmacytoma will have other systemic signs of multiple myelomas, such as this patient [5,6]. Isolated cases may be treated with radiation alone, surgery being reserved for symptoms from mass effect and chemotherapy for systemic illness, both of which were present in this patient.

Plasmacytomas are a collection of abnormal white blood cells. They most commonly form in bone but can also form in soft tissues, most commonly the head, neck, and respiratory and gastrointestinal systems [3]. Although unclear, a common thought is that intracranial plasmacytomas develop in the skull prior to spreading to soft tissue [9]. They can be solitary or be associated with multiple myeloma. Patients diagnosed with solitary plasmacytoma can go on to develop multiple myeloma, with one study showing a median onset time of 21 months and 45% developing multiple myeloma at five years [3]. In a case series, all 22 cases of suprasellar plasmacytomas exhibited bony invasion, and all but one had an intact anterior pituitary function [4]. As mentioned before, definitive diagnosis is dependent upon biopsy, as clinical presentation and imaging findings for plasmacytoma are very similar to pituitary adenoma [4]. Due to structural similarities between plasmacytoma and pituitary adenomas on light microscopy, immunohistological staining is often necessary [4,10].

Treatment for plasmacytoma and multiple myeloma requires collaboration between sub-specialties including medical oncologists, radiation oncologists, surgeons as well as hematologists/oncologists specializing in stem cell transplants. When a solitary plasmacytoma is diagnosed, surgery or radiation is often the treatment of choice.

**Figure 4:**
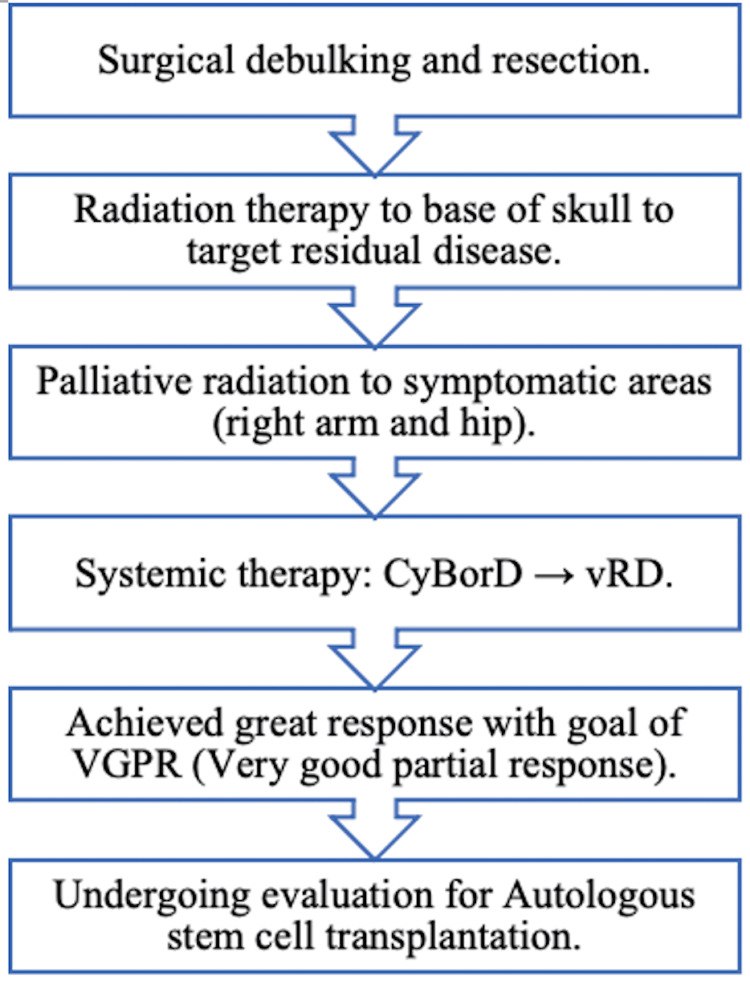
Step-by-step treatment plan for the patient’s multiple myeloma CyBorD: cyclophosphamide, bortezomib, and dexamethasone; vRD: bortezomib, lenalidomide, and dexamethasone.

However, as our patient was diagnosed with multiple myeloma, his care was targeted in a multi-disciplinary fashion (Figure 4). Immediately after his diagnosis, he underwent max safe resection of the plasmacytoma allowing for debulking and symptomatic benefit. He underwent radiation and received a total of 20 Gy to the base of the skull using 18 MV via three-dimensional conformal radiation therapy (3D CRT). The specific dose of radiation is variable based on location and size. Consensus on the optimal dose of radiation is still under debate but is generally around 40 Gy for definitive treatment for solitary plasmacytomas [11].

However, if there is evidence of disease elsewhere or evidence of end-organ damage, systemic therapy is often required. With the introduction of proteasome inhibitors and multi-drug regimens to combat resistance, the overall survival of patients has increased dramatically over the past few years [12]. The combination of bortezomib, lenalidomide, and dexamethasone has become the therapy of choice for patients who are candidates for aggressive treatments. Following the induction phase of treatment, patients should be evaluated for autologous stem cell transplant, as this increases progression-free and overall survival [13]. The patient was initially started on CyBorD due to logistical challenges but was quickly switched over to the primary choice of treatment of vRD.

Quick efforts in the management of this patient’s care allowed us to attain a challenging diagnosis and start treatment immediately. Unfortunately, his vision in the right eye was compromised but we were able to achieve a VGPR or near-complete response per IWMG criteria, allowing the patient to be considered for autologous stem cell transplantation.

Despite efforts in using multi-drug regimens to combat resistance, relapses remain common in the course of multiple myeloma and plasmacytomas. Careful evaluation of a patient's symptoms, laboratory work, and imaging if necessary should be done at periodic intervals.

## Conclusions

Presentation of plasmacytomas can be variable among patients and diagnosis requires careful evaluation of symptoms with laboratory and pathological evaluation. Plasmacytomas are a collection of malignant plasma cells. If systemic symptoms/spread is present, multiple myeloma is diagnosed. Intracranial lesions can present as an initial presentation of plasmacytoma. Biopsy-dependent diagnosis and immunohistochemical staining are often necessary due to structural similarities to adenomas on microscopy. In conclusion, we would like to highlight the rareness of plasmacytomas in the suprasellar region and would like to emphasize the importance of considering such a diagnosis in the differential diagnosis of intracranial lesions as a delay in diagnosis can lead to a delay in treatment and can be detrimental to overall outcomes.

## References

[1] (2022). Cancer stat facts: myeloma. https://seer.cancer.gov/statfacts/html/mulmy.html.

[2] Kumar SK, Rajkumar SV, Dispenzieri A (2008). Improved survival in multiple myeloma and the impact of novel therapies. Blood.

[3] Ozsahin M, Tsang RW, Poortmans P (2006). Outcomes and patterns of failure in solitary plasmacytoma: a multicenter Rare Cancer Network study of 258 patients. Int J Radiat Oncol Biol Phys.

[4] Sinnott BP, Hatipoglu B, Sarne DH (2006). Intrasellar plasmacytoma presenting as a non-functional invasive pituitary macro-adenoma: case report & literature review. Pituitary.

[5] DiDomenico J, Ampie L, Choy W, Lamano JB, Oyon DE, Kesavabhotla K, Bloch O (2018). Sellar plasmacytomas masquerading as pituitary adenomas: a systematic review. J Clin Neurosci.

[6] Lee J, Kulubya E, Pressman BD, Mamelak A, Bannykh S, Zada G, Cooper O (2017). Sellar and clival plasmacytomas: case series of 5 patients with systematic review of 65 published cases. Pituitary.

[7] Famini P, Maya MM, Melmed S (2011). Pituitary magnetic resonance imaging for sellar and parasellar masses: ten-year experience in 2598 patients. J Clin Endocrinol Metab.

[8] Silverstein A, Doniger DE (1963). Neurologic complications of myelomatosis. Arch Neurol.

[9] Clarke E (1954). Cranial and intracranial myelomas. Brain.

[10] Sautner D, Saeger W, Lüdecke DK (1993). Tumors of the sellar region mimicking pituitary adenomas. Exp Clin Endocrinol.

[11] Reed V, Shah J, Medeiros LJ (2011). Solitary plasmacytomas: outcome and prognostic factors after definitive radiation therapy. Cancer.

[12] Durie BG, Hoering A, Sexton R (2020). Longer term follow-up of the randomized phase III trial SWOG S0777: bortezomib, lenalidomide and dexamethasone vs. lenalidomide and dexamethasone in patients (Pts) with previously untreated multiple myeloma without an intent for immediate autologous stem cell transplant (ASCT). Blood Cancer J.

[13] Vesole DH, Tricot G, Jagannath S (1996). Autotransplants in multiple myeloma: what have we learned?. Blood.

